# Severity of Lower Urinary Tract Symptoms among Middle Aged and Elderly Nigerian Men: Impact on Quality of Life

**DOI:** 10.1155/2016/1015796

**Published:** 2016-06-19

**Authors:** Patrick Temi Adegun, Philip Babatunde Adebayo, Peter Olufemi Areo

**Affiliations:** ^1^Department of Surgery, College of Medicine, Ekiti State University, Ekiti State 36001, Nigeria; ^2^Department of Medicine, Ladoke Akintola University of Technology, Ogbomoso, Oyo State, Nigeria

## Abstract

*Objectives*. To compare the severity of LUTS among middle aged and elderly Nigerian men and determine the influence of LUTS severity on QoL.* Methods*. This cross-sectional study was conducted among new patients presenting with LUTS attending Urology clinic between 2011 and 2015. Assessment of symptoms was based on IPSS and bother score completed by the eligible subjects on the same day of their clinic visits.* Results*. Four hundred patients were studied comprising 229 middle aged and 171 elderly men. Interquartile range (IQR) of IPSS scores for men <65 years and those ≥65 years was 14.0 (16.0) and 19 (15.0), respectively (*p* < 0.001). Mild LUTS was significantly associated with best, good, and poor quality of life while moderate LUTS was associated with poor QoL. Severe LUTS was significantly associated with all the categories of QoL (Best-Worst). Among the cohort of subjects with poor QoL, elderly patients had a significantly higher median IPSS score (*p* < 0.05).* Conclusions*. There is no level of severity of LUTS in which patients' QoL is not impaired although mild symptomatology may be associated with better QoL and severe symptomatology with poor QoL. Careful attention to QoL may help identify patients who require early and prompt treatment irrespective of the IPSS.

## 1. Introduction

Olmsted County study, one of the largest longitudinal studies conducted in America, investigated age as one of many sociodemographic characteristics that may predict the incidence of LUTS/BPH. Also, age was reported to be one of the most reliable risk factors for the progression of LUTS/BPH. Its influence is greater than those of other sociodemographic characteristics [1].

Aging as a process has been discovered to be associated with the development of various, sometimes distressing, symptoms of different organ systems in the body including the genitourinary tract [2].

Besides, European and Korean EPIC studies have estimated that about 2/3rd of LUTS is present in the middle aged men whereas other researchers reported that the conditions leading to LUTS are among the most prevalent diseases of the elderly, with serious impairment of quality of life [QoL] [1, 3–15].

However, as men age, there is an increasing prevalence of Lower Urinary Tract Symptoms (LUTS) but no clear difference in the impact of LUTS on the QoL between the middle aged and the elderly men [16].

Therefore, there is the need to differentiate between the effects of severity of LUTS on the quality of life of middle aged and the elderly men. Comparison of impact of LUTS in these men is important to improve the management of LUTS in men, some of whom are still in the working class and the bread winners for their families.

The objective of this study was to compare the severity of LUTS among middle aged and elderly Nigerian men attending the Urology clinic at Ekiti State University Teaching Hospital, South Western Nigeria. We also aimed to determine the influence of LUTS severity on the QoL of these men.

## 2. Methods

### 2.1. Settings

This was a comparative cross-sectional study carried out on all new patients who presented to the Urology clinic of Ekiti State University Teaching Hospital, Ado-Ekiti, South Western Nigerian from July 1, 2011, to June 30, 2015.

### 2.2. Selection Criteria and Data Collection

The inclusion criteria were male sex; 40 years aged and above; voluntary participation; and understanding and signing the consent form. The exclusion criteria were previous open prostatectomy; acute diseases such as sepsis syndrome, cardiovascular events, and trauma; surgeries or hospitalizations during the preceding month; and uncompensated chronic diseases.

A questionnaire containing sociodemographics and concurrent medical conditions including history of alcohol ingestion and 8-item International Prostate Symptoms Score (IPSS) (English version was used because official national language in Nigeria is English) was completed by the eligible subjects on the same day of their clinic visits. The IPSS is a reliable and widely used instrument since 1991, and it is based on the answers to seven questions concerning urinary symptoms and one question concerning quality of life [17]. Each question concerning urinary symptoms allows the patient to choose one out of six answers indicating increasing severity of the particular symptom. The answers are assigned points from 0 to 5. The total score ranges from 0 to 35 (asymptomatic to very symptomatic). The questions refer to the following urinary symptoms: (i) incomplete emptying, (ii) frequency, (iii) intermittency, (iv) urgency, (v) weak stream, (vi) straining, and (vii) nocturia. Question  8 measures patient's perceived quality of life (QoL) which comprises seven answers from 0 to 6. The quality of life or level of satisfaction of LUTS of patients was represented by seven grades: “no problem” (0 point  =  very satisfied), “I'm all right” (1 point), “somewhat satisfied” (2 points), “half satisfied, half dissatisfied” (3 points), “somewhat dissatisfied” (4 points), “distressed” (5 points), and “I can't stand it” (6 points  = very dissatisfied).

Self-administration of questionnaires was preferred, but face-to-face interviews were conducted whenever the participants presented with visual deficits, illiteracy, or semi-illiteracy that would preclude them from proper completion of the questionnaire. Thirteen (13) participants had their questionnaire interviewer administered. Trained medical staff conducted the interviews in private rooms, which lasted an average of 30 minutes.

### 2.3. Ethical Issues

The study was conducted in accordance with Declaration of Helsinki (as revised in Edinburgh 2000). Written informed consent was obtained from all participants before participation in the study.

Ethical approval was obtained from Ethical Research Committee of the Ekiti State University Teaching Hospital, Ado-Ekiti, Nigeria.

### 2.4. Statistical Analysis

For statistical analysis, the subject's demographic and clinical variables were summarized and presented as frequencies and percentages for categorical variables while numerical data were summarized as means and standard deviation when normally distributed and median with interquartile range (IQR) when skewed. IPSS was categorized into mild (0–7), moderate (8–19) and severe symptoms (20–35) while quality of life (QoL) defined by bother score was categorized as best for BS = 0-1; good for BS = 2-3; poor for BS = 4-5; and worst for BS = 6.

Patients demographic and clinical data were summarized and presented as frequencies and percentages while the chi-squared test was used to test differences between the middle aged (<65 years) and elderly men (≥65 years). Skewed continuous variables were summarized as median (interquartile range) while Mann-Whitney *U* nonparametric test was used to test the differences in the median values between middle aged and elderly subjects. Chi-squared test was used to analyse the difference between quality of life categories (bother score) and severity of LUTS (defined by IPSS). To detect the significant group, the chi-squared test was followed by a multiple pairwise comparison test with adjustment of the *p* values. All statistical analyses were performed using SPSS version 20.0 (SPSS, Chicago, Illinois). A *p* value < 0.05 was considered to be statistically significant while adjusted *p* value of <0.0042 was considered significant for the post hoc pairwise comparison.

## 3. Results

Table 1 showed that while elderly men were either married or widowers or single or divorced men could be found among the middle aged. This finding was statistically significant. In addition, majority of the elderly men were retired. While majority of the middle aged cohort were overweight, normal weight and obese men were more common among the elderly. This was also significant. Among the comorbidities studied, systemic hypertension and diabetes mellitus were more prevalent in the elderly, while alcohol consumption was prevalent in the middle aged men.  Table 2 showed that the severity of LUTS was significantly worse among subjects of 65 years and above (*p* < 0.05).

Table 3 showed the quality of life of patients with LUTS in relation to the severity of the symptoms where the IPSS score was categorized. There was a statistically significant relationship between the severity of LUTS and the QoL of the subjects.

Table 4 shows the results of post hoc multiple pairwise comparisons of the categories of the contingency Table 3 after the chi-squared test has rejected the null hypothesis of equality of proportions of subjects across the cells. The table showed the pairwise proportions among the multiple cross-classifications that led to the statistical significance observed in Table 3 after adjusting the *p* value to 0.0042. Mild symptomatic was significantly associated with best, good, and poor quality of life while moderate symptomatic was associated with poor QoL. Severe symptomatic was significantly associated with the entire categories of QoL (Best-Worst).

**Figure 1:**
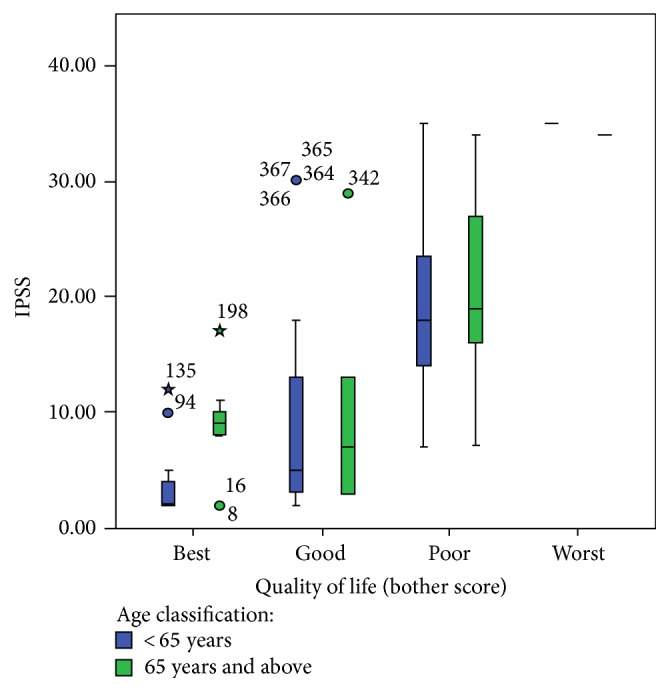
Showing quality of life versus age in years.

Figure 1 showed the IPSS scores among the subjects with different perceived QoL according to the age group. Among the cohort with best quality of life, the elderly patients had a statistically significant median IPSS score (*p* > 0.05). No statistical difference exists between the median IPSS scores of the cohort who had good QoL (*p* > 0.05). Among the cohort of subjects with poor QoL, elderly patients had a statistically significantly higher median IPSS score (*p* > 0.05).

## 4. Discussion

We sought to compare the severity of symptoms on the QoL of middle aged and elderly Nigerian men. This result showed that QoL was affected by any category of symptoms (mild to severe) across the age. It was demonstrated in this study that even mild symptomatology of IPSS could be associated with poor quality of life whereas some severe symptomatology was associated with good QoL (*p* < 0.05). Therefore patients' health seeking behaviour might have been influenced by their QoL rather than the severity of the IPSS score. This finding is in agreement with the report of Finkelstein et al. that discovered that an individual's perceived impact on health-related quality of life might be a determinant for patients to seek medical advice [18].

In addition, the study also showed that severity of IPSS increases with age (*p* < 0.001). This is similar to the findings of Engström et al. that reported that severity of symptoms increases with age [19].

More importantly, among the cohort with best quality of life, the elderly patients had a statistically significant higher median IPSS score (*p* < 0.05). This might be due to high prevalence of hypertension in the elderly which could increase IPSS coupled with higher prevalence of alcohol consumption in the middle aged which might cause lower IPSS in them. However, a longitudinal study on the volume of alcohol consumption in this environment is necessary to corroborate this assertion. This is in line with Suh et al., Lu, and Mo that reported a similar protective effect of light-moderate alcohol consumption on IPSS [20, 21].

Furthermore, this study showed that elderly men had higher frequency of poor QoL (*p* < 0.001). This is similar to the findings of Welch et al. that men with moderate and severe LUTS identified in a large US cohort had a poorer health status in several important quality of life dimensions [14]. It is important that these elderly men are adequately assessed for appropriate therapy. Surgical treatments have been strongly favoured for moderate to severe symptomatic patients and watchful waiting or conservative measures indicated for patients with mild complaints [22–24].

Recognition of significant deterioration of QoL among mild, moderate, and severe LUTS patients is an evidence for the need of treatment (as opposed to watchful and conservative approaches) and justification for early treatment irrespective of IPSS score. This is in consonance with Finkelstein et al. that noted that the IPSS quality of life question had a larger effect size than all the other measures suggesting that this single-item measure may have high sensitivity to differentiate subgroups [18].

## 5. Conclusion

From the foregoing, it might be better to use the QoL as determinant of the choice of treatment rather than the IPSS scores alone for prompt treatment of LUTS and improved clinical outcome.

## 6. Limitation of the Study

However, criticisms have been made regarding the poor standardization of QoL scales and the frequent inappropriate use of the term QoL. The use of a one-item scale to assess general QoL (the IPSS-QoL question, called “bother score”) and the misinterpretation of QoL as synonymous to symptom-control or perceived general health or functional status are the most frequent reasons for such criticisms. Besides, because this study was hospital-based, it may not be a true random sample of men with LUTS in the general population. It is difficult to eliminate selection bias.

Furthermore, we did not evaluate for depressive symptomatology and other psychosocial contributors to QoL perception. However, the measure of QoL in this study is a disease specific instrument with psychometric properties well fitted to measure QoL as it relates to urinary symptoms.

## Figures and Tables

**Table 1:** Demographic characteristics of the study population.

Variables	Age < 65 years; 229 (%)	Age ≥ 65 years; 171 (%)	Test statistics	*p* value
Marital status				<0.001
Single	23 (10)	0 (0)	35.36	
Married	193 (84.3)	166 (97.1)		
Divorced	13 (5.7)	0 (0)		
Widower	0 (0)	5 (2.9)		
Occupation			77.62	<0.001
Public servant	106 (46.3)	19 (11.1)		
Business	31 (13.5)	45 (26.3)		
Retired	39 (17.0)	79 (46.2)		
Others	53 (23.1)	28 (16.4)		
BMI				
Underweight	0 (0)	1 (0.6)	11.21	0.011
Normal	67 (29.3)	71 (41.5)		
Overweight	126 (55.0)	67 (39.2)		
Obese	36 (15.7)	32 (18.7)		
Comorbidities				
Hypertension	120 (52.4)	115 (67.3)	8.91	0.003
Diabetes mellitus	18 (7.9)	20 (11.7)	1.67	0.196
Alcohol	91 (39.7)	36 (21.1)	11.05	0.001
Ultrasound findings				
Normal size	52 (22.7)	2 (1.2)	11.95	0.003
Enlarged and benign	161 (70.3)	160 (93.6)		
Suspected cancer	16 (7.0)	9 (5.3)		
DRE			18.50	<0.001
Normal sized prostate	35 (15.3)	9 (5.3)		
Enlarged and benign	178 (77.7)	161 (94.2)		
Suspicious lesion	16 (7.0)	1 (0.6)		
Diagnosis			5.46	0.141
BPH	181 (79.0)	141 (82.5)		
CaP	23 (10.0)	22 (12.9)		
Urethral stricture	6 (2.6)	2 (1.2)		
Others for example OAB	19 (8.3)	6 (3.5)		

CaP = cancer of the prostate; OAB = overactive bladder.

**Table 2:** IPSS, voiding, and storage subscores among the age group.

Variables	Age < 65 years	Age ≥ 65 years	Mann-Whitney *U* test	*p* value
	Median (IQR)	Median (IQR)		
IPSS	14.0 (16.0)	19.0 (15.0)	15435.0	<0.001
Voiding symptoms	7.0 (12.0)	9.0 (10.0)	16041.0	0.002
Storage symptoms	7.0 (7.0)	9.0 (5.0)	15804.5	0.001

**Table 3:** Showing the quality of life of the subjects in relation to the severity of LUTS symptoms.

IPSS severity	Bother score				Chi-squared test	*p* value
	Best QoL	Good QoL	Poor QoL	Worst QoL		
	*n* (%)	*n* (%)	*n* (%)	*n* (%)		
Mildly symptomatic	26 (68.4)	42 (55.3)	12 (4.3)	0 (0)	175.75	<0.001
Moderately symptomatic	10 (26.3)	21 (27.6)	136 (49.3)	0 (0)		
Severely symptomatic	2 (5.3)	13 (17.1)	128 (46.4)	10 (100)		

Total	36 (100)	76 (100)	276 (100)	10 (100)		

**Table 4:** Pairwise comparison of IPSS severity and quality of life of patients with LUTS.

Pair compared	Chi-squared test	*p* value
Mildly symptomatic versus best QoL	60.84	<0.001^*∗*^
Mildly symptomatic versus good QoL	72.25	<0.001^*∗*^
Mildly symptomatic versus poor QoL	136.89	<0.001^*∗*^
Mildly symptomatic versus worst QoL	2.56	0.109
Moderately symptomatic versus best QoL	4.00	0.046
Moderately symptomatic versus good QoL	7.84	0.005
Moderately symptomatic versus poor QoL	21.16	<0.001^*∗*^
Moderately symptomatic versus worst QoL	7.29	0.007
Severely symptomatic versus best QoL	19.36	<0.001^*∗*^
Severely symptomatic versus good QoL	17.64	<0.001^*∗*^
Severely symptomatic versus poor QoL	25.00	<0.001^*∗*^
Severely symptomatic versus worst QoL	16.81	<0.001^*∗*^

Adjusted *p* value = 0.0042.

^*∗*^statistically significant.
